# Evaluating the Diagnosis of Malnutrition Based on Global Leadership Initiative on Malnutrition (GLIM) Criteria in Community-Dwelling Older Adults (Singapore Longitudinal Aging Study)

**DOI:** 10.3390/nu16223823

**Published:** 2024-11-07

**Authors:** Phoo Pyae Sone Win, Denise Qian Ling Chua, Xinyi Gwee, Shiou Liang Wee, Tze Pin Ng

**Affiliations:** 1 Formerly Health and Social Sciences, Singapore Institute of Technology, Singapore 828608, Singapore; phuupyaesone@gmail.com; 2 Health Services and Systems Research, Duke-NUS Medical School, Singapore 169857, Singapore; 3 Formerly Department of Psychological Medicine, Yong Loo Lin School of Medicine, National University of Singapore, Singapore 119228, Singapore; dcql1213@gmail.com (D.Q.L.C.); gweexinyi@gmail.com (X.G.); pcmngtp@gmail.com (T.P.N.); 4 Geriatric Education and Research Institute, Ministry of Health, Singapore 169854, Singapore; 5 SR Nathan School of Human Development, Singapore University of Social Sciences, Singapore 599494, Singapore

**Keywords:** malnutrition, mortality, quality of life, ENIGMA, MNA

## Abstract

Background: A minority of studies using the GLIM criteria for malnutrition diagnosis have performed formal empirical validation. Objectives: To evaluate the concurrent and predictive validity of GLIM criteria with and without prior screening among community-dwelling older adults in Singapore. Method: In the Singapore Longitudinal Aging Study (SLAS-2, *n* = 2477), malnutrition was diagnosed using single-step and two-step GLIM procedures using the Mini Nutritional Assessment Short Form (MNA-SF) and Elderly Nutritional Index for Geriatric Malnutrition Assessment (ENIGMA) for initial screening. Criterion validity was evaluated using MNA-Full Form (MNA-FF) as reference malnutrition diagnosis. Prognostic validity was evaluated using logistic and Cox regression analyses with respect to impaired quality of life (QoL) and 10-year mortality. Results: GLIM malnutrition with and without MNA-SF or ENIGMA screening showed significant associations with known clinical correlates; single-step GLIM malnutrition: sensitivity = 80%, specificity = 83%; two-step MNA-SF-GLIM malnutrition: sensitivity = 80%, specificity = 85%; two-step ENIGMA-GLIM malnutrition: sensitivity = 74%, specificity = 88%; positive predictive values of around 20% and negative predictive values above 98%. Cohen’s kappa values of agreement were uniformly low (0.26 to 0.32). All showed significant associations with about 50% increased odds of impaired QoL and 10-year mortality, adjusted for age, sex, ethnicity, education levels, and housing type, with the ENIGMA-GLIM malnutrition showing the highest risk estimates. Compared to MNA-FF malnutrition prevalence of 4.1%, GLIM-based malnutrition increased prevalence (14.6% to 19.7%) estimates. Conclusions: The GLIM criteria showed good construct and criterion validity. It increased the number of individuals diagnosed with malnutrition. The agreement between diagnoses of malnutrition was low. Diagnostic and prognostic accuracy vary with the screening instrument used. Early identification of malnutrition using appropriate tools can provide opportunities to delay or prevent the risk of important adverse outcomes such as impaired QoL and mortality.

## 1. Introduction

The prevalence of malnutrition in community-dwelling older adults ranged from 0.8 to 24.6% in different parts of the world [1]. A scoping review of Singapore local studies reported that 2.8–31.5% of the elderly in the community were found to be malnourished, depending on the screening and assessment tools that were used [2]. The identification of malnutrition across diverse healthcare settings has long been problematic because of a lack of common terminology and standard criteria for its diagnosis. Recent efforts have established a consensus for the definition of malnutrition based on the concept that it reflects the catabolism of lean tissues and diminished function resulting from decreased energy and protein intake and/or assimilation and/or inflammation. In line with this, the Global Leadership Initiative on Malnutrition (GLIM) has proposed operational criteria for the diagnosis of malnutrition based on the minimum of one phenotypic criterion (significant weight loss OR low body mass index OR low muscle mass) and one etiologic criteria (reduced food intake/assimilation OR disease burden/inflammation) [3]. The GLIM criteria, as it is based on expert opinions, require empirical validation [4]. According to a review of 96 studies that have employed the GLIM criteria, only a small minority of studies have evaluated at least one aspect of the construct and criterion (concurrent or predictive) validity of the GLIM criteria [5].

The GLIM criteria may be used alongside current validated screening and assessment tools for identifying patients already malnourished and at risk of malnutrition or assessing risk factors predisposing to malnutrition. A two-step process uses a screening tool to identify individuals with high risk or probability of malnutrition, followed by a comprehensive clinical assessment to establish malnutrition diagnosis based on the GLIM criteria. Short of a ‘gold standard’ clinical assessment to diagnose malnutrition by a qualified health professional, ‘hybrid’ nutritional measurement tools, such as the Subjective Global Assessment (SGA) [6,7] or the Mini-Nutritional Assessment Full Form (MNA-FF) [8,9], which are able to provide more comprehensive information than mere screening tools, are often used as surrogate diagnostic tools.

Given the different malnutrition-related risks—such as worse clinical outcomes (increased overall mortality, longer hospital stays, and higher risk of readmission), frailty, and sarcopenia [2]—together with the rapidly aging global population [10], it is crucial to have validated tools for the nutritional assessment of the elderly. Therefore, in this study, we evaluated the concurrent and predictive validity of the GLIM criteria for malnutrition diagnosis in a population cohort of community-dwelling older adults. Prevalent cases of malnutrition were identified in the whole study cohort. As recommended, in a two-step process, we used the Short Form of the MNA (MNA-SF) and the ENIGMA as initial screening tools for the tentative identification of older adults at risk of malnutrition or malnutrition followed by the application of the GLIM criteria for malnutrition diagnosis (MNA-SF-GLIM malnutrition and ENIGMA-GLIM malnutrition, respectively). At the same time, we employed a single-step procedure to ‘diagnose’ malnutrition using standalone GLIM, MNA-FF, and ENIGMA (using an appropriate higher threshold cutoff as validated in previous studies).

To evaluate the concurrent criterion validity of the GLIM criteria for malnutrition diagnosis, we used the MNA-FF as a quasi-gold standard measure. MNA-FF, which is a brief, standardized tool and has been validated previously, is deemed appropriate to be used as a semi-gold standard tool and is considered a preferred tool for criterion validation [4]. The predictive validity of GLIM-based malnutrition (GLIM malnutrition, ENIGMA-GLIM malnutrition, and MNA-SF-GLIM malnutrition) was evaluated with respect to clinical outcomes in terms of prevalent impaired quality of life (QoL) and 10-year mortality risks.

## 2. Methods

The Singapore Longitudinal Aging Study (SLAS) is a population-based observational study that followed two cohorts (SLAS-1 and SLAS-2) of community-dwelling older adults who were aged 55 years and above in Singapore. Detailed information on the survey methodology, baseline, and follow-up data collection were described in previous publications [11]. The study was conducted in accordance with the Declaration of Helsinki and the Belmont Report. All procedures involving human subjects were approved by the Institutional Review Board of the National University of Singapore (NUS-IRB 04-140, approval date 17 June 2005). All participants gave their informed consent.

The present study is a post-hoc analysis of data extracted in the SLAS-2 study with 2477 participants who had complete baseline data for identifying GLIM malnutrition. The SLAS-2 cohort comprised residents living in the South-West and South-Central regions of Singapore recruited for baseline assessment between 2010 and 2013 with subsequent follow-up at 3–5 yearly intervals. Available questionnaire, clinical, and blood testing data at baseline were used to determine malnutrition status based on MNA-SF-GLIM, ENIGMA-GLIM, and GLIM criteria, using appropriate cutoffs for identifying nutritional risk and malnutrition. The reporting of this study follows the STROBE guidelines [12] for observational studies, and the completed checklist is available as Supplementary Materials (Supplementary File S1).

### 2.1. GLIM Criteria

Phenotypic criteria: Low BMI was defined by the Asian criteria proposed by the GLIM consensus report: <18.5 kg/m^2^ if younger than 70 years or <20 kg/m^2^ if older than 70 years [3]. Weight loss was determined by unintended weight loss > 10 lb/4 kg in the past 6 months [11]. Muscle mass was considered reduced in the presence of low calf circumference value, using cutoff values of <33 cm for females and <34 cm for males, respectively, according to the Asian Working Group for Sarcopenia [13].

Etiologic criteria: Reduced food intake was determined by a question on the presence of illness/condition that changes the kind/amount of food eaten [11]. Inflammation was determined by elevated levels of C-reactive Protein (CRP) ≥ 10 mg/L (Table 1).

### 2.2. Mini Nutritional Assessment (MNA)

Participants were scored on the 6 items of the MNA-SF (diet intake, weight loss, mobility, psychological stress or acute disease, neuropsychological problems, and BMI) [14]. The derivation of MNA scores using SLAS available data and the classification of participants for malnutrition risk and malnutrition were described in detail in a previous study [11]. Participants were at risk of malnutrition (or malnourished) if the MNA screening score was ≤11 points.

The reference ‘gold standard’ diagnosis of malnutrition was made using the Full Form of the MNA (MNA-FF), which has an additional 12 question items for those who score ≤11 points on the MNA-SF screening tool [8]. The additional questions include questions on the phenotypic manifestations (such as mid-arm and calf circumference and self-assesses nutritional and health status), risk factors (such as meal frequency, protein intake through dairy products, fish or meat, legumes, or eggs), and fruit and vegetable consumption. Individuals who scored <17 points on the MNA-FF were categorized as having malnutrition.

### 2.3. Elderly Nutritional Indicators for Geriatric Malnutrition Assessment (ENIGMA)

The full details for the development and validation of the ENIGMA were described in previous publications [15,16]. The ENIGMA uses four questionnaire-based nutritional indicators: physically unable to shop, cook and/or feed myself, take 3 or more different drugs a day, tooth or mouth problem causes difficulty eating and few fruit or vegetables (less than 2 portions per day) as well as four blood/serum markers (low hemoglobin, low albumin, low total cholesterol, low lymphocyte count). According to a previous study [15], malnutrition risk was categorized by ENIGMA indexes as low (0–1), moderate (2–3), and high (4+). In this study, participants were considered to be at risk of malnutrition (or malnourished) if the ENIGMA score was ≥1 point. An ENIGMA score of ≥4 was used to define malnutrition.

### 2.4. GLIM-Based Malnutrition

Using the single-step procedure, participants in the whole cohort were classified as malnourished if they met at least one phenotypic criterion (weight loss, low BMI, or reduced muscle mass) and one etiologic criterion (reduced food intake or disease burden or inflammation). Malnutrition identified as such was labeled as GLIM malnutrition.

In the two-step procedure, we incorporated MNA-SF and ENIGMA as initial screening tools, followed by GLIM criteria for the diagnosis of malnutrition. Participants were screened positive for nutritional risk using an MNA-SF score cutoff ≤11 and an ENIGMA score ≥ 1, according to previous publications. Malnutrition diagnosed by GLIM criteria following MNA-SF screening was labeled as MNA-SF-GLIM malnutrition, and malnutrition by GLIM criteria following ENIGMA screening was labeled as ENIGMA-GLIM malnutrition (Table 2).

### 2.5. Malnutrition-Related Adverse Health Outcomes

Malnutrition-related health outcome measures were impaired QoL and 10-year mortality risk. We evaluated the association between these adverse health outcomes and malnutrition, as identified by one-step GLIM, MNA-SF-GLIM, and ENIGMA-GLIM. The assessment of QoL was performed using the Medical Outcomes Study SF-12 Physical Component Score (PCS) [17]. The SF-12 is a patient-based outcome measure assessing the effect of health on a person’s daily life and is often applied as a quality-of-life measure. In this study, participants were asked to rate their health in accordance with the SF-12 Physical Component Summary Score (12 questions including general health perception, physical functioning, role-physical, bodily pain, and general health). A PCS score below the lowest quartile in the distribution was used to define impaired QoL in this study.

### 2.6. Mortality Follow-Up

The participants’ date of death from the start of the study until 31 March 2021 was determined by linking their unique National Registration Identity Card (NRIC) number with computerized records of deaths in the National Death Registry at the National Disease Registry Office (NDRO) of the Ministry of Health, Singapore.

### 2.7. Confounding Variables

Age, sex, ethnicity, housing type (an indicator of socioeconomic status), and the presence of 3 or more chronic diseases such as diabetes, hypertension, coronary heart disease, asthma, end-stage renal disease ESRD, depression (geriatric depression scale GDS score ≥ 5), and cognitive impairment (Mini-Mental State Examination, MMSE score ≤23) were analyzed as potential confounding variables.

### 2.8. Statistical Analysis

The descriptive data for continuous variables were presented as either mean ± standard deviation (SD) or median with upper and lower quartiles, depending on whether the data were parametric or non-parametric. Categorical variables were presented as number (*n*) and their relative frequency in percentage. Independent t-test for continuous parametric data, the Mann–Whitney test for continuous non-parametric data, and the chi-square test were used to assess the association between nutrition status and participants’ general characteristics. The distribution of the data was assessed for normality using the Shapiro–Wilk normality test.

To assess criterion validity of GLIM-based diagnosis of malnutrition (single-step GLIM, ENIGMA-GLIM, and MNA-SF-GLIM) in relation to MNA-FF as reference measure for malnutrition, sensitivity, specificity, positive predictive value (PPV), negative predictive value (NPV), kappa values, and Youden index were estimated.

To assess the association of single-step GLIM, ENIGMA-GLIM, and MNA-SF-GLIM diagnoses of malnutrition with its adverse health outcomes, we used logistic regression models to estimate odds ratios (ORs) and 95% confidence intervals (CI). Model 1 was an unadjusted model, and OR estimates were adjusted for age, sex, ethnicity, and housing type (socio-demographic) in model 2. In model 3, OR estimates were adjusted for model 2 plus the presence of 3 or more chronic diseases, functional dependency at baseline, GDS depression, and MMSE cognitive impairment at baseline. Cox proportionate hazard regression analyses were performed to estimate hazard ratios (HRs) and 95% CI of 10-year mortality occurrence.

Survival analysis with the Kaplan–Meier method and the log-rank test were used to compare survival rates between individuals with and without malnutrition (defined as the presence or absence of GLIM malnutrition, ENIGMA-GLIM malnutrition, and MNA-SF-GLIM malnutrition) on time-to-event data, censored at the date of death or on 31 March 2021.

Data were analyzed using R statistical software (version R 4.2.3).

## 3. Results

### 3.1. Characteristics of the Study Group

A total of 2477 participants with complete baseline data for classification of GLIM criteria were included in the study. The median age of the study participants was 65.0 (Q1–Q2 60.0–71.0) and 62.8% of participants were women (Table 3). During the period of follow-up for mortality, 378 (15.3%) participants died, and the median survival years was 9.9.

Table 2 shows the prevalence of each phenotypic and etiologic criteria of GLIM malnutrition. The most common phenotypic criterion was low muscle mass (30.6%), and the most common etiologic criterion was reduced food intake (34.8%). The prevalence of single-step GLIM malnutrition was 19.7%. Using the appropriate validated screening thresholds, the prevalence of participants screened positive was 63.5% by MNA-SF and 63.0% by ENIGMA. The prevalence of GLIM-based malnutrition diagnosed by a two-step procedure was 18.3% by MNA-SF-GLIM and 14.6% by ENIGMA-GLIM. The prevalence of the reference MNA-FF malnutrition was 4.9%.

Malnutrition identified using a single-step procedure (GLIM malnutrition) was significantly associated with older age, lower education and housing statuses, ethnicity, poor MMSE performance score, higher GDS score, higher prevalent co-morbidity, and poor functional mobility compared to the non-malnourished group, as well as lower BMI, lesser muscle mass, more prevalent weight loss, and reduced food intake. There was no significant association with sex. MNA-SF-GLIM malnutrition and ENIGMA-GLIM malnutrition also showed very similar patterns of significant associations with these nutrition-related risk factors and clinical correlates.

### 3.2. Concurrent Criterion Validity

Using MNA-FF malnutrition as a reference criterion, GLIM malnutrition, MNA-SF-GLIM malnutrition, and ENIGMA-GLIM malnutrition showed moderate to high sensitivity (74–80%) and high specificity (83–88%). All showed high NPV of 98–99% but low PPV of 20–25%. Cohen’s kappa values of agreement were uniformly low (0.26 to 0.32) (Table 4).

### 3.3. Associations with Impaired QoL

Single-step GLIM, MNA-SF-GLIM, and ENIGMA-GLIM malnutrition were all significantly associated with impaired QoL after adjustment for age, sex, ethnicity, education levels, and housing type (Table 5). Among them, ENIGMA-GLIM malnutrition showed the highest estimated odds ratio (OR = 1.47, 95% CI = 1.14–1.89). There were no significant associations after adjustment for clinical variables known to be associated with poor QoL (three or more chronic diseases, functional dependency at baseline, GDS depression, and MMSE cognitive impairment).

### 3.4. Associations with 10-Year Mortality

**Figure 1:**
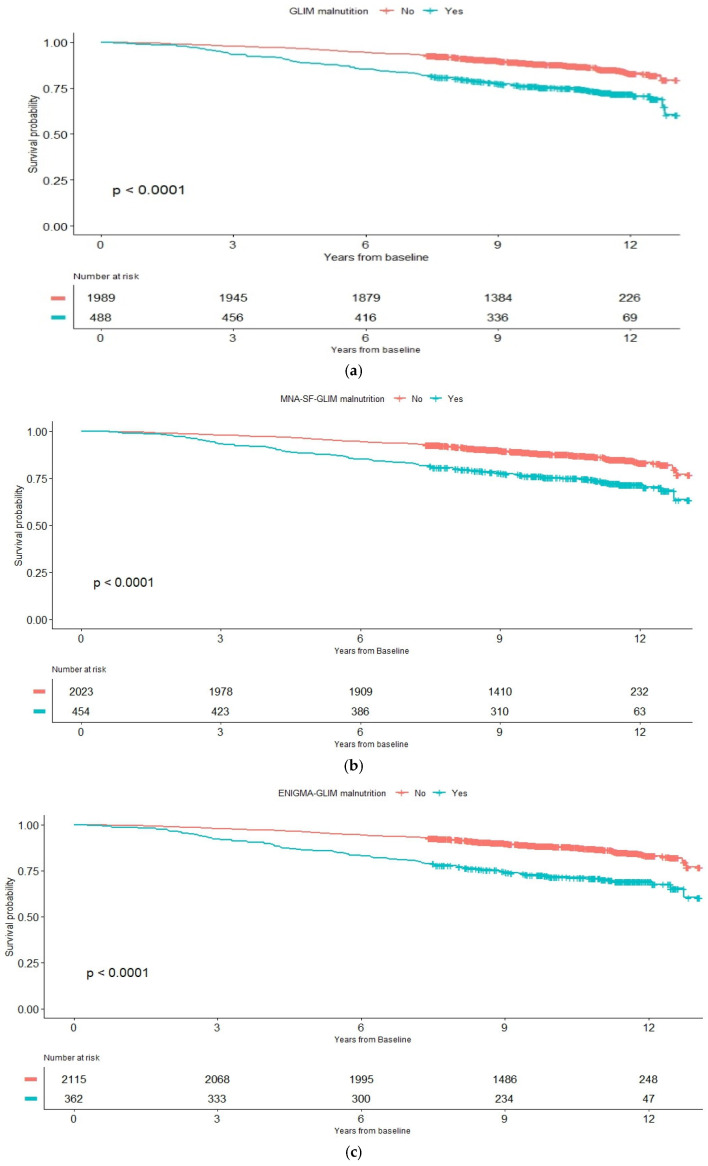
Kaplan–Meier curve survival analysis on (**a**) GLIM malnutrition, (**b**) MNA-SF-GLIM malnutrition, and (**c**) ENIGMA-GLIM malnutrition.

As shown in Table 6, Figure 1, all three GLIM-based measures of malnutrition were significantly associated with around 50% increased risk of 10-year mortality after adjustment for age, sex, ethnicity, education levels, and housing type. ENIGMA-GLIM malnutrition showed comparatively higher mortality HR (1.55, 95% CI = 1.23–1.96). With further adjustment for three or more chronic diseases, functional dependency at baseline, GDS depression, and MMSE cognitive impairment, the HRs remained significant, showing at least a 35% increased mortality rate (for MNA-SF-GLIM malnutrition). EMIGMA-GLIM malnutrition showed comparatively highest mortality HR at 1.42 (95% CI = 1.12–1.80).

## 4. Discussion

In summary, our findings suggested that the diagnosis of malnutrition based on the GLIM criteria with or without initial MNA-SF or ENIGMA screening individually showed good construct and criterion validity. They showed significant associations with known risk factors and clinical correlates, good accuracy in discriminating between individuals with malnutrition and those without, and good predictive accuracy in prognosticating impaired QoL and 10-year mortality.

Validity. We observed in this Asian population study that single-step GLIM malnutrition showed good sensitivity (80%) and specificity (83%) when evaluated against MNA-FF malnutrition as a reference comparator. The findings from our study were congruent with the results of a recent meta-analysis of 20 studies, including 10 studies in community settings, which found good diagnostic accuracy in studies that evaluated it against SGA as the reference standard (sensitivity, 0.80; specificity, 0.81) [18]. In this study, the GLIM criteria also significantly predicted long-term mortality; this had also been documented in previous studies of community-dwelling older adults as well [19,20]. In this population setting, the NPV is very high (99%), but the PPV is low (20%), indicating that 80 out of 100 individuals with positive GLIM-based screening results do not actually have malnutrition (false positive). For the purpose of screening and diagnosis of malnutrition, in which the risks of harm or moral distress from false positive and false negative tests are low compared to devastating diagnoses such as cancer, and follow-up tests are inexpensive and easily and quickly performed, this level of PPV and NPV is tolerable and can be adjusted by altering the stringency of the screening test [21].

Comparatively, there were marginal differences in diagnostic and prognostic accuracy among the three GLIM-based diagnoses of malnutrition with or without screening. In the two-step process, MNA-SF screening resulted in marginal improvement in specificity to 85% with no loss of sensitivity, whereas the ENIGMA screening resulted in greater improvement in specificity to 88% but with some loss of sensitivity to 74%. The variation in diagnostic and prognostic accuracy based on the different screening methods used may partly be explained by the different compositions of malnutrition criteria by different screening tools. In MNA-SF, participants were assessed based on the presence of acute disease and neuropsychological problems. This could result in lower malnutrition prevalence for community-dwelling older adults who are in better health than their hospitalized counterparts. The ENIGMA was developed with the impacts and contributions of its components towards mortality, and, therefore, screening with it may have resulted in comparatively higher mortality HR.

All three GLIM-based diagnoses of malnutrition significantly predicted impaired QoL and 10-year mortality, but the ENIGMA-GLIM malnutrition appears to show greater predictive validity. All GLIM-based diagnoses of malnutrition, however, showed low concordance with the quasi-gold standard MNA-FF diagnosis of malnutrition. The low kappa statistics also showed that they did not necessarily identify the same individuals with malnutrition. This has also been observed in previous studies [6,22]. The loss of agreement could suggest that tools may not be interchangeable or that there could be a need for further refinement of the GLIM criteria to better align with MNA-FF. Further validity studies utilizing MNA-FF as a reference in different populations may be necessary.

Prevalence. The application of GLIM criteria with or without initial screening appeared to diagnose more individuals with malnutrition, giving higher estimates of malnutrition prevalence compared to the prevalence of MNA-FF malnutrition. The greater number of individuals with GLIM-based malnutrition was largely driven by the high prevalence (over 30%) of those with reduced muscle mass and those who reported reduced food intake. The prevalence of malnutrition using the quasi-gold standard MNA-FF assessment tool was 4.1% (alternately, the prevalence of ENIGMA malnutrition, using a high threshold cutoff of ≥4 was 8.9%). The prevalence of GLIM-based malnutrition without screening (19.7%) and screening with MNA-SF (18.3%) were higher. The prevalence of ENIGMA-GLIM malnutrition was somewhat lower (14.6%), consistent with previous observations that the prevalence of malnutrition based on the GLIM criterion varies with the choice of nutrition screening tools [9,23].

The 18.3% prevalence of GLIM malnutrition after initial MNA-SF screening in our study is within the range reported in prior studies of community-dwelling elderly, for example, in Belgium (23.4%) [20], Turkey (24.5%) [24], and Spain (12.6%) [19]. The lower prevalence in Spain could be largely attributed to a healthier population, with almost half (45.2%) of the elderly with no co-morbidity and a higher mean BMI (28.25 ± 5.62) in their malnourished group compared to ours (BMI 24.5 ± 4.1).

We compared our results to those in a similar study of Chinese individuals but in a hospital setting, which also used MNA-SF screening prior to GLIM diagnosis and MNA-FF as a reference standard [9]. That study showed even higher sensitivity (90.5%) and specificity (86.4%), as well as greater concordance (Kappa = 0.629). The prevalence of the risk of malnutrition was 49.7% (versus 63.5% in this study) after MNA-SF screening, and malnutrition after the application of GLIM criteria was 27.8% (versus 18.3% in this study). Given the difference in hospital and community settings in the two studies, the prevalence of MNA-SF-GLIM malnutrition in the two studies may be regarded as broadly compatible.

The ENIGMA is a new screening tool that we have recently reported to have strong construct, concurrent, and predictive validity in community-dwelling cohorts [15,16]. Owing to its brevity and ease of use, it serves well as a screening tool for the identification of GLIM malnutrition. It also possesses high content validity in capturing the core phenotypic and etiologic facets of malnutrition, as it is able to provide more comprehensive nutritional information than a screening tool. For this reason, it appears to show better discriminant and predictive accuracy in the diagnosis of GLIM malnutrition following initial ENIGMA screening in this study. However, this needs to be further investigated.

The current GLIM criteria allow different and multiple combinations of phenotypic and etiologic components for the diagnosis of malnutrition, and it was also noted that the GLIM criteria require further standardization, especially for cutoff values and various combinations of phenotypic and etiologic components [4]. Such variations may limit the generalizability of our study. However, the phenotypic and etiologic characteristics used in this study were inexpensive and easily accessible in different healthcare systems and regions. Therefore, our study can easily be replicated and compared with future prospective studies.

## 5. Conclusions

The GLIM criteria showed good construct and criterion validity against previously validated nutritional assessment tools. For community-dwelling older adults, early identification of malnutrition using appropriate tools may offer opportunities to delay or prevent poor QoL and 10-year mortality risks.

Strengths and limitations. We performed this validation study in a large population cohort with long-term follow-up of mortality outcomes. Another strength is that we used the full MNA assessment questionnaire. This is an acceptable reference for criterion validation in place of an in-depth nutritional assessment completed by a trained nutritional expert. In this regard, MNA-SF screening for GLIM malnutrition may be criticized for providing a more favorable estimate of criterion validity. ENIGMA screening is less subject to criterion contamination, and its diagnostic performance relative to MNA-SF screening prior to GLIM criteria application could possibly be greater. We used the calf muscle circumference as it is a simple and validated alternative measure of reduced muscle mass, feasible for use in the community setting. The use of bioimpedance or imaging-based analysis is preferred, but they are difficult to perform even in clinical settings, resulting in considerable missing data (79%) in one study [5]. Although the GLIM criteria with MNA-SF and the ENIGMA screening were shown in this study to have good criterion validity in the community setting, more studies are needed to confirm its validity in hospitals and other community settings. Additionally, because this is an observational study, the statistically significant association between ethnicity, socioeconomic status, and clinical correlates are to be carefully interpreted, and further research may be necessary to better understand the underlying factors contributing to these associations.

## Figures and Tables

**Table 1:** Phenotypic and etiologic criteria of GLIM malnutrition.

Phenotypic Criteria	Weight Loss	Unintended Weight Loss > 10 lb/4 kg in the Past 6 Months	
		or	
	Low BMI	<18.5 kg/m^2^ if younger than 70 years, <20 kg/m^2^ if 70 years and older.	
		or	
	Reduced muscle mass	Calf-circumference < 33 cm (female), <34 cm (male)	
		AND	
Etiologic criteria	Reduced food intake	“Do you have an illness/condition that reduces the kind or amount of food eaten?”	
		or	
	Disease burden/inflammation	CRP ≥ 10 mg/L	

**Table 2:** Prevalence of phenotypic and etiologic components of GLIM criteria, malnutrition according to different GLIM criteria, ENIGMA malnutrition, and MNA-FF malnutrition in SLAS-2 cohort (*n* = 2477).

Criteria	Prevalence, *n* (%)
Phenotypic Components	
Weight loss (Unintended weight loss > 10 lbs/4 kg last 6 months)	43 (1.7)
Low BMI Asia criteria	172 (6.9)
- <18.5 kg/m^2^ if <70 years	60 (2.4)
- <20 kg/m^2^ if >70 years	112 (4.5)
Reduced muscle mass (Calf circumference)	758 (30.6)
- Female (<33 cm)	485 (19.6)
- Male (<34 cm)	273 (11)
Etiologic Components	
Reduced food intake	863 (34.8)
C-reactive Protein (CRP) ≥ 10 mg/L	617 (24.9)
Screening thresholds	
ENIGMA ≥ 1 points	1560 (63.0)
MNA-SF ≤ 11 points	1574 (63.5)
Malnutrition diagnosis	
GLIM malnutrition	488 (19.7)
MNA-SF-GLIM malnutrition	454 (18.3)
ENIGMA-GLIM malnutrition	362 (14.6)
ENIGMA malnutrition, single-step ENIGMA with high threshold cutoff (≥4)	220 (8.9)
MNA-FF malnutrition, single-step MNA-FF with high threshold cutoff (<17)	121 (4.9)

GLIM malnutrition, single-step GLIM criteria without initial risk screening. ENIGMA-GLIM malnutrition, participants at risk identified by the ENIGMA (screened ‘at risk of malnutrition (or malnourished)’ if ENIGMA score is ≥1 point). MNA-SF-GLIM malnutrition, participants at risk identified by the MNA-SF (screened ‘at risk of malnutrition (or malnourished)’ if the MNA-SF screening score is ≤11 points).

**Table 3:** Baseline characteristics of non-malnourished and malnourished study participants as identified by single-step GLIM criteria (GLIM malnutrition) (*n* = 2477).

		GLIM Malnutrition		*p*	MNA-SF-GLIM Malnutrition		*p*	ENIGMA-GLIM Malnutrition		*p*
	Total	Yes (*n* = 488)	No (*n* = 1989)		Yes (*n* = 454)	No (*n* = 2023)		Yes (*n* = 362)	No (*n* = 2115)	
Age (years), median (Q1–Q3)	65.0 (60.0–71.0)	68.5 (62–75)	65 (60–71)	<0.0001	69 (62–75)	65.0 (60–71)	<0.0001	70.0 (63–75)	65.0 (60–71)	<0.0001
Female, *n* (%)	1556 (62.8)	303 (62.1)	1253 (63.0)	0.7498	282 (62.1)	1274 (63.0)	0.7723	217 (59.9)	1339 (63.3)	0.2439
Education, *n* (%): None	463 (23.6)	115 (23.6)	348 (17.5)	<0.0001	108 (23.8)	355 (17.6)	<0.0001	94 (26.0)	369 (17.4)	<0.0001
Primary	1065 (45.9)	224 (45.9)	841 (42.3)		209 (46.0)	856 (42.3)		165 (45.6)	900 (42.6)	
Secondary and above	949 (30.5)	149 (30.5)	800 (40.2)		137 (30.2)	812 (40.1)		103 (28.4)	846 (40.0)	
Housing, *n* (%): 1–2 room flat	512 (20.7)	143 (29.3)	369 (18.6)	<0.0001	132 (29.1)	380 (18.8)	<0.0001	117 (32.3)	395 (18.7)	<0.0001
3-room flat	694 (28.1)	150 (30.7)	544 (27.4)		142 (31.3)	552 (27.4)		104 (28.7)	590 (28.0)	
4+ rooms and others	1266 (51.2)	195 (40.0)	1071 (54.0)		180 (39.6)	1086 (53.8)		141 (39.0)	1125 (53.3)	
Ethnicity, *n* (%): Chinese	2176	407 (83.4)	1769 (88.9)	0.0006	381 (83.9)	1795 (88.7)	0.004	300 (82.9)	1876 (88.7)	0.0004
Malay	178	41 (8.4)	137 (6.9)		37 (8.1)	141 (7.0)		30 (8.3)	148 (7.0)	
Indian/others	123	40 (8.2)	83 (4.2)		36 (7.9)	87 (4.3)		32 (8.8)	91 (4.3)	
MMSE score, median (Q1–Q3)	29.0 (27–30)	28 (26–29)	29 (27–30)	<0.0001	28 (26–29)	29 (27–30)	<0.0001	28 (26–29)	29 (27–30)	<0.0001
GDS score, median (Q1–Q3)	0 (0–1)	1(0–1)	0 (0–1)	<0.0001	1 (0–1)	0 (0–1)	<0.0001	1 (0–1)	0 (0–1)	<0.0001
Co-morbidity (≥3 chronic diseases), *n* (%)	1116 (45.1)	275 (56.4)	841 (42.3)	<0.0001	266 (58.6)	850 (42.0)	<0.0001	223 (61.6)	893 (42.2)	<0.0001
Weight loss, *n* (%)	43 (1.7)	24 (4.9)	19 (1.0)	<0.0001	24 (5.3)	19 (0.9)	<0.0001	21 (5.8)	22 (1.0)	<0.0001
BMI, median (Q1–Q3)	24.2 (21.8–26.8)	21.9 (20–24)	25.1 (22–27)	<0.0001	21.5 (20–24)	24.7 (22–27)	<0.0001	21.8 (20–24)	24.6 (22–27)	<0.0001
Calf-circumference (cm), median (Q1–Q3)	34.5 (32.0–36.5)	31.0 (30.0–32.0)	35.0 (34.0–37.0)	<0.0001	31.0 (30.0–32.0)	35.0 (33.5–37.0)	<0.0001	31.0 (30.0–32.0)	35.0 (33.0–37.0)	<0.0001
Reduced food intake, *n* (%)	863 (34.8)	360 (73.8)	503 (25.3)	<0.0001	360 (79.3)	503 (24.9)	<0.0001	280 (77.3)	583 (27.6)	<0.0001
Follow-up years, median (Q1–Q3)	9.9 (8.7–11.3)	10.6 (8.3–11.6)	9.8 (8.7–11.3)	<0.0001	10.5 (8.2–11.6)	9.9 (8.7–11.3)	<0.0001	10.1 (7.9–11.6)	9.9 (8.8–11.3)	<0.0001

GLIM malnutrition, single-step GLIM criteria without initial risk screening. ENIGMA-GLIM malnutrition, participants at risk identified by the ENIGMA (screened ‘at risk of malnutrition (or malnourished)’ if ENIGMA score is ≥1 point). MNA-SF-GLIM malnutrition, participants at risk identified by the MNA-SF (screened ‘at risk of malnutrition (or malnourished)’ if the MNA-SF screening score is <11 points); MMSE, Mini-Mental State Examination; GDS score, Geriatric Depression Scale; BMI, body mass index.

**Table 4:** Concordance of malnutrition GLIM criteria with and without initial screening against MNA-FF malnutrition.

Reference: MNA-FF Malnutrition	GLIM Malnutrition	MNA-SF-GLIM Malnutrition	ENIGMA-GLIM Malnutrition
Youden Index	0.63	0.65	0.62
Sensitivity (%)	80%	80%	74%
Specificity (%)	83%	85%	88%
PPV	20%	21%	25%
NPV	99%	99%	98%
Kappa (95% CI)	0.26 (0.22–0.31)	0.28 (0.23–0.33)	0.32 (0.26–0.37)

MNA-FF malnutrition, single-step MNA-FF with high threshold cutoff (<17). GLIM malnutrition, single-step GLIM criteria without initial risk screening; MNA-SF-GLIM malnutrition, participants at risk identified by the MNA-SF (screened ‘at risk of malnutrition (or malnourished)’ if the MNA-SF screening score is ≤11 points); ENIGMA-GLIM malnutrition, participants at risk identified by the ENIGMA (screened ‘at risk of malnutrition (or malnourished)’ if ENIGMA score is ≥1 point); PPV, positive predictive value; NPV, negative predictive value

**Table 5:** Associations of malnutrition defined by different malnutrition tools with impaired quality of life (QoL).

Malnutrition Criteria	*n* = 2477	Impaired Quality of Life (*n* = 597)		Model 1 (Unadjusted)			Model 2 ^a^			Model 3 ^b^		
		*n*	%	OR	95% CI		OR	95% CI		OR	95% CI	
GLIM malnutrition	Yes (*n* = 488)	159	32.6	1.71	1.37	2.12 ***	1.41	1.12	1.77 **	1.17	0.91	1.49
	No (*n* = 1989)	438	22.0	1			1			1		
MNA-SF-GLIM malnutrition	Yes (*n* = 454)	147	32.4	1.68	1.34	2.10 ***	1.37	1.08	1.74 ***	1.09	0.85	1.41
	No (*n* = 2023)	450	22.2	1			1			1		
ENIGMA-GLIM malnutrition	Yes (*n* = 362)	126	34.8	1.86	1.46	2.37 ***	1.47	1.14	1.89 **	1.16	0.88	1.53
	No (*n* = 2115)	471	22.3	1			1			1		

^a^ Model 2: adjusted for age, sex, ethnicity, education levels, and housing type. ^b^ Model 3: adjusted for age, sex, ethnicity, education levels, housing type, 3 or more chronic diseases, functional dependency at baseline, GDS depression, and MMSE cognitive impairment. GLIM malnutrition, single-step GLIM criteria without initial risk screening; MNA-SF-GLIM malnutrition, participants at risk identified by the MNA-SF (screened ‘at risk of malnutrition (or malnourished)’ if the MNA screening score is ≤11 points); ENIGMA-GLIM malnutrition, participants at risk identified by the ENIGMA (screened ‘at risk of malnutrition (or malnourished)’ if ENIGMA score is ≥1 point); ** *p* < 0.01; *** *p* < 0.001.

**Table 6:** Mortality rates associated with GLIM malnutrition, MNA-SF-GLIM malnutrition, and ENIGMA-GLIM malnutrition (*n* = 2477).

		Exposed	Follow-Up	Mortality			Model 1 (Unadjusted)			Model 2 ^a^		Model 3 ^b^		
		*n*	Person-years (p-y)	*n* = 378	Per 1000 p-y	HR	95% CI		HR	95% CI		HR	95% CI	
GLIM malnutrition	Yes	488	4638.1	130	28.0	2.14	1.73	2.65 ***	1.51	1.21	1.88 ***	1.41	1.12	1.76 **
	No	1989	19,308	248	12.8	1			1			1		
MNA-SF-GLIM malnutrition	Yes	454	4292.8	121	28.2	2.13	1.71	2.64 ***	1.47	1.18	1.84 ***	1.35	1.07	1.70 *
	No	2023	19,654	257	13.1	1			1			1		
ENIGMA-GLIM malnutrition	Yes	362	3341.9	108	32.3	2.44	1.95	3.05 ***	1.55	1.23	1.96 ***	1.42	1.12	1.80 **
	No	2115	20,605	270	13.1	1			1			1		

^a^ Model 2: adjusted for age, sex, ethnicity, education levels, and housing type. ^b^ Model 3: adjusted for age, sex, ethnicity, education levels, housing type, 3 or more chronic diseases, functional dependency at baseline, GDS depression, and MMSE cognitive impairment. GLIM malnutrition, single-step GLIM criteria without initial risk screening; MNA-SF-GLIM malnutrition, participants at risk identified by the MNA-SF (screened ‘at risk of malnutrition (or malnourished)’ if the MNA screening score is ≤11 points); ENIGMA-GLIM malnutrition, participants at risk identified by the ENIGMA (screened ‘at risk of malnutrition (or malnourished)’ if ENIGMA score is ≥1 point); * *p* < 0.05; ** *p* < 0.01; *** *p* < 0.001.

## Data Availability

The datasets used and/or analyzed during the current study are available from the corresponding author upon reasonable request.

## Supplementary Materials

The following supporting information can be downloaded at https://www.mdpi.com/article/10.3390/nu16223823/s1; Supplementary File S1: STROBE Statement—Checklist of items that should be included in reports of cohort studies.
